# *HOXB1* Is a Tumor Suppressor Gene Regulated by miR-3175 in Glioma

**DOI:** 10.1371/journal.pone.0142387

**Published:** 2015-11-13

**Authors:** Liang Han, Dehua Liu, Zhaohui Li, Nan Tian, Ziwu Han, Guang Wang, Yao Fu, Zhigang Guo, Zifeng Zhu, Chao Du, Yu Tian

**Affiliations:** 1 Department of Neurosurgery, China-Japan Union Hospital of Jilin University, Changchun, Jilin, China; 2 Department of Cell Biology, College of Life Science, Zhejiang Chinese Medical University, Hangzhou, Zhejiang, China; 3 Department of Interventional Therapy, the First Hospital of Jilin University, Changchun, Jilin, China; University of Pécs Medical School, HUNGARY

## Abstract

The *HOXB1* gene plays a critical role as an oncogene in diverse tumors. However, the functional role of HOXB1 and the mechanism regulating HOXB1 expression in glioma are not fully understood. A preliminary bioinformatics analysis showed that HOXB1 is ectopically expressed in glioma, and that HOXB1 is a possible target of miR-3175. In this study, we investigated the function of HOXB1 and the relationship between HOXB1 and miR-3175 in glioma. We show that HOXB1 expression is significantly downregulated in glioma tissues and cell lines, and that its expression may be closely associated with the degree of malignancy. Reduced HOXB1 expression promoted the proliferation and invasion of glioma cells, and inhibited their apoptosis in vitro, and the downregulation of HOXB1 was also associated with worse survival in glioma patients. More importantly, HOXB1 was shown experimentally to be a direct target of miR-3175 in this study. The downregulated expression of miR-3175 inhibited cell proliferation and invasion, and promoted apoptosis in glioma. The oncogenicity induced by low HOXB1 expression was prevented by an miR-3175 inhibitor in glioma cells. Our results suggest that HOXB1 functions as a tumor suppressor, regulated by miR-3175 in glioma. These results clarify the pathogenesis of glioma and offer a potential target for its treatment.

## Introduction

Glioma is the most frequent primary malignant tumor of the adult central nervous system (CNS), and is characterized by high morbidity and poor survival [1–3]. Despite advances in the diagnosis and appropriate systemic therapies for glioma, including surgery, radiotherapy, and chemotherapy, there has been very little improvement in the clinical outcomes of patients with this cancer, and more than 70% of patients succumb to the disease within 2 years of diagnosis [4–5]. Studies have shown that the survival of glioma patients depends on the tumor type and the grade of the malignancy [6]. Accumulating research has shown that several biological and molecular factors are involved in the development, progression, and metastasis of glioma [7]. Therefore, it is essential to identify novel molecular markers that can efficiently predict its prognosis and provide targets for molecular therapies.

The HOX genes encode a highly conserved family of transcription factors, containing a 60-amino-acid, helix-turn-helix DNA-binding domain, that play important roles in development, regulating numerous processes, including apoptosis, receptor signaling, differentiation, motility, and angiogenesis [8]. HOXB1 is reported to be differentially expressed in abnormal development and malignancy, indicating that the altered expression of HOXB1 is important in both oncogenesis and tumor suppression. For example, the suppression of HOXB1 expression in pancreatic cancer is sufficient to promote metastasis [9]. HOXB1 also reduces cell growth and proliferation and induces apoptosis and cell differentiation in acute myeloid leukemia, depending on the downregulation of some tumor-promoting genes, in parallel with the upregulated expression of apoptosis- and differentiation-related genes [10]. The HOXB1-regulated expression of COL5A2, which is involved in the focal adhesion pathway, correlates with the carcinogenesis of endometrial cancer [11], and HOXB1 also regulates HXR9, which causes the apoptosis of breast cancer cells [12]. The relationships between the HOX genes and glioma have been investigated for a long time [13–14], but the expression and function of HOXB1 in glioma are still unclear. Therefore, in this study, we first investigated whether the expression of HOXB1 is abnormal in glioma, whether it correlates with patient survival, and the function of HOXB1 in oncogenesis.

The transcription of the HOX genes is regulated by several proteins and RNAs, including the trithorax group proteins, polycomb repressor complex 2 (PRC2), HOTAIR, and microRNAs (miRNAs) [8]. It is well known that miRNAs play key roles in diverse biological processes, including cell differentiation, apoptosis, proliferation, and migration, through their interaction with one or more target genes [15–16]. We investigate whether the expression of HOXB1 is also regulated by miRNAs, and whether the tumorigenic role of HOXB1 is affected by miRNAs in glioma. Computer-assisted bioinformatic analyses were performed to predict the putative miRNAs that bind the *HOXB1* 3′-untranslated region (3′-UTR), and the miRNA they all predicted was miR-3175. In this study, we suggest that *HOXB1* functions as a tumor suppressor gene in glioma and that the expression of *HOXB1* is regulated by miR-3175. These results extend our understanding of the molecular mechanism of the tumorigenesis of glioma and offer a potential target for glioma therapies.

## Materials and Methods

### Human tissue specimens and cell lines

Human glioma specimens and normal tissues were obtained from the Department of Neurosurgery, China-Japan Union Hospital of Jinlin University. This study obtained Institutional Review Board (IRB) approval regarding the use of human samples for experimental studies from the Ethics Committees of the China-Japan Union Hospital of Jilin University and written informed consent was obtained from all the patients or their families. We also obtained written informed consent from guardians on behalf of the minors enrolled in the study. Forty glioma tissue specimens were taken from patients who underwent surgery between September 2010 and October 2012. All glioma specimens were histologically classified and graded according to the World Health Organization Classification of Tumors of the Central Nervous System guideline. Complete clinical data were acquired at the initial diagnosis and follow-ups. To maintain the integrity of the data, we adhered to the principles expressed in the Declaration of Helsinki. Eight nonneoplastic brain tissue specimens, for use as controls, were obtained from patients with severe traumatic brain injury, who had required partial resection of their brain tissue. All human tissue specimens were freshly resected at surgery and immediately frozen in liquid nitrogen until analysis. Overall survival (OS) and progression-free survival (PFS) were assessed and calculated from the follow-up data of the glioma patients. OS was defined as the period from the date of the pathological diagnosis to death; PFS was defined as the period from the date of the pathological diagnosis to glioma progression, presenting as new neurological symptoms. We downloaded mRNA expression microarray data for 212 gliomas from the Chinese Glioma Genome Atlas (CGGA) data portal (http://www.cgga.org.cn/) and 180 gliomas from the Gene Expression Omnibus website (http://www.ncbi.nlm.nih.gov/geo/; GSE4290).

Human brain glioma cell lines A172, U251, and U87 and an astrocyte cell line, HA1800 (used as the control), were purchased from the Type Culture Collection of the Chinese Academy of Sciences (Shanghai, China). The cells were cultured in Dulbecco’s modified Eagle’s medium (Gibco-BRL, Carlsbad, CA, USA) containing 10% fetal bovine serum (FBS; Gibco-BRL), 100 U/ml penicillin, and 100 μg/ml streptomycin in a humidified atmosphere of 5% CO_2_ at 37°C.

### RNA preparation and quantitative reverse transcription–PCR (qRT-PCR)

Total RNA was extracted from the human tissue specimens and glioma cells with TRIzol Reagent (Invitrogen, Carlsbad, CA, USA), according to the manufacturer’s instructions. The first-strand complementary DNAs (cDNAs) were synthesized with the HiFi-MMLV cDNA Kit (CWBiotech, Beijing, China), according to the manufacturer’s instructions. The traditional oligo(dT) primer method and the stem-loop-specific primer method were used to verify the expression levels of *HOXB1* and miR-3175, respectively. qRT-PCR was performed with the StepOnePlus™ Real-Time PCR System (Applied Biosystems, Foster, CA, USA) using the UltraSYBR Mixture (CWBiotech, Beijing, China). *HOXB1* expression was normalized to that of the endogenous control housekeeping gene *ACTB*, encoding β-actin, and miR-3175 expression was normalized to that of U6. The PCR cycling parameters were 95°C for 10 min, followed by 40 cycles of 95°C for 15 s, 58°C for 30 s, and 72°C for 30 s. The qRT-PCR results were evaluated with the 2^–ΔΔCt^ method. The following primers were used in the PCR reactions: HOXB1 forward: 5′-GGTATGCTCCTGCCGCCTGCA-3′ and HOXB1 reverse: 5′-ATCAGCATAGGCCGGTGCAA-3′; miR-3175 stem-loop primer 5′-GTCGTATCCAGTGCAGGGTCCGAGGTATTCGCACTGGATACGACACGTCACT-3′, miR-3175 forward: 5′-GATACTCACGGGGAGAGAACGCAG-3′, miR-3175 reverse: 5′-GTGCAGGGTCCGAGGT-3′; β-actin forward: 5′-GGCACCACACCTTCTACAAT-3′ and β-actin reverse: 5′-GTGGTGGTGAAGCTGTAGCC-3′; and U6 forward: 5′-CTCGCTTCGGCAGCACA-3′ and U6 reverse: 5′-AACGCTTCACGAATTTGCGT-3′.

### Protein extraction and western blot analysis

Total proteins were extracted from all the cultured cells with RIPA buffer (Boster, Wuhan, China) containing 1 mM phenylmethanesulfonyl fluoride, according to the manufacturer’s protocol. The protein concentrations were determined with a BCA Protein Assay Kit (KeyGEN, Shanghai, China), and the proteins were then separated with sodium dodecyl sulfate polyacrylamide gel electrophoresis and transferred to polyvinylidene difluoride (PVDF) membranes. The PVDF membranes were blocked overnight at 4°C with 5% bovine serum albumin (BSA; Amresco, OH, USA). After the membranes were washed with Tris-buffered saline-Tween 20 at room temperature, they were incubated sequentially with diluted (1:500) primary antibodies directed against HOXB1 (Santa Cruz Biotechnology, Santa Cruz, CA, USA) and other proteins, including procaspase-3, p53, and cytochrome C (1:200; Boster). The membranes were washed again and incubated with a horseradish-peroxidase-conjugated secondary antibody (1:2000; Santa Cruz Biotechnology). β-Actin was used as the endogenous reference control.

### Immunohistochemical analysis

Immunohistochemical staining was performed to determine the expression and subcellular localization of the HOXB1 protein in the human glioma specimens and nonneoplastic brain tissues. The tissues were embedded in paraffin and cut into 4 μm thick sections, which were deparaffinized and rehydrated, and then retrieved with heat-induced epitope retrieval. Endogenous peroxidase was inhibited with 3% hydrogen peroxide (H_2_O_2_) and nonspecific antigens were blocked with 5% BSA. The slides were then incubated overnight at 4°C with a primary antibody directed against HOXB1 (1:100; Santa Cruz Biotechnology). After the slides were washed three times with phosphate-buffered saline (PBS) for 5 min each at room temperature, a biotinylated secondary antibody (1:1000) was added and the samples incubated for 1 h. After the slides were rinsed three times with PBS, the streptavidin-biotin–peroxidase complex (1:100) was applied to them. The reaction products were visualized with 3,3′-diaminobenzidine tetrahydrochloride (DAB), according to the manufacturer’s instructions. All tissue sections were viewed with a Nikon Eclipse 80i microscope (Nikon, Tokyo, Japan) fitted with a camera. The images were analyzed with the ImagePro Plus 6.0 software.

### Bioinformatic prediction

A predictive search for miRNAs targeting *HOXB1* was performed using the programs miRanda (http://www.microrna.org/), DIANA TOOLS (http://diana.imis.athena-innovation.gr), and TargetScan (http://www.targetscan.org/).

### Luciferase assay

The 3′-UTR of *HOXB1* was synthesized, annealed, and inserted into the *Xba*I and *Aha*III sites of the pmirGLO luciferase reporter vector (Promega, Madison, WI, USA), downstream from the stop codon of the luciferase gene. The sequence of wild-type (WT) *HOXB1* complementary to the binding site of miR-3175 in the 3′-UTR (TCTCCCC) was replaced with a mutated (MUT) *HOXB1* sequence (TGAGGCC). The constructs were confirmed with DNA sequencing. Glioma U87 cells were cotransfected with pmirGLO-WT-HOXB1 or pmirGLO-MUT-HOXB1, the pRL-TK *Renilla* plasmid (Promega), and equal amounts of the negative control, miR-3175 mimic or miR-3175 mimic. The pRL-TK *Renilla* luciferase reporter vector was used as the internal control. After the cells were incubated for 48 h, the luciferase activity was measured with the Dual Luciferase Reporter Assay Kit (Beyotime, Haimen, China).

### Oligonucleotides and transfection

The miR-3175 mimic, the miR-3175 inhibitor, and the corresponding negative controls (NC) were synthesized and purified by GenePharma, (Shanghai, China). The sequences were: miR-3175 mimic: 5′-ACGUCACUGCGUUCUCUCCCCG-3′; miR-3175 inhibitor: 5′-CGGGGAGAGAACGCAGUGACGU-3′; miR-3175 mimic NC: 5′-UACUTCGAACCAACCAGCGUUACT-3′; and miR-3175 inhibitor NC: 5′-CATUCCUUGTUGUGUAAUCCUUTT-3′. Three small interfering RNAs (siRNAs) targeting *HOXB1* (si-HOXB1) and an siRNA negative control (si-NC) were designed and synthesized by GenePharma, Si-HOXB1-1 forward: 5′-GATCCCCTTCTTAGAGTACCCACTTTCAAGAGAAGTGGGTACTCTAAGAAGGTTTTTTA-3′, si-HOXB1-1 reverse: 5′-AGCTTAAAAAACCTTCTTAGAGTACCCACTTCTCTTGAAAGTGGGTACTCTAAGAAGGG-3′; si-HOXB1-2 forward: 5′-GATCCCTTCTCAGTACTACCCTCTTTCAAGAGAAGAGGGTAGTACTGAGAAGTTTTTTA-3′, si-HOXB1-2 reverse: 5′-AGCTTAAAAAACTTCTCAGTACTACCCTCTTCTCTTGAAAGAGGGTAGTACTGAGAAGG-3′; si-HOXB1-3 forward: 5′-GATCCCTCAATGAAACACAGGTCATTCAAGAGATGACCTGTGTTTCATTGAGTTTTTTA-3′, si-HOXB1-3 reverse: 5′-AGCTTAAAAAACTCAATGAAACACAGGTCATCTCTTGAATGACCTGTGTTTCATTGAGG-3′; si-NC forward 5′-AATGGGTAAACCGTTAAACAGTCCCTAAGAGATAAATGGCCTCGAATCCTATC-3′, si-NC reverse: 5′-CCCTGAAGGAAGAAAGGATTCGCAAACGTATCAAGGGATCGGTTAACTTAACC-3′. One day before transfection, the glioma cells were plated at a density of 3–5 × 10^5^ cells/well in six-well plates so that the cells were grown to 70%-80% confluence at the time of transfection. The cells were transfected with the oligonucleotides described above using Lipofectamine 2000 (Invitrogen, Carlsbad, CA, USA), according to the manufacturer’s protocol. The glioma cells were harvested 48–72 h after the initial transfection for further analysis.

### Cell proliferation assay

The viability of the glioma cells transfected with specific oligonucleotides was determined with the 3-(4,5-dimethylthiazol-2-yl)-2,5-diphenyltetrazolium bromide (MTT; Amresco) uptake method. Briefly, after the cells were incubated for 24 h, they were transfected with specific oligonucleotides, and seeded in a 96-well plate (2 × 10^3^/well). The cells were incubated for another 48 h in medium containing 10% FBS. MTT solution (10 μl; 5 mg/ml) was added to each well and the plate was incubated for 4 h at 37°C in an incubator. After the medium was removed, the purple formazan crystals were dissolved in 150 μl/well dimethyl sulfoxide (DMSO; Amresco) for 30 min. The absorbance of the formazan was measured at 490 nm on a SpectraMax M3 microplate reader (Molecular Devices, USA).

### In vitro invasion assay

The Transwell assay and scratch wound assay were used to determine the invasion capacity of the transfected cells in vitro. In the Transwell assay, glioma cells transfected with the appropriate oligonucleotides were incubated for 48 h, and 3 × 10^5^ cells/ml were transferred into the top of the Matrigel™-coated invasion chamber (BD Biosciences, San Jose, USA). The upper chamber was loaded with 200 μl of serum-free medium and the lower chamber was loaded with 700 μl of medium containing 10% FBS. After incubation for 18 h in an incubator at 37°C, the upper-chamber medium was cautiously removed, the glioma cells were fixed in 4% paraformaldehyde for 15 min and stained with Giemsa for 20 min, and the noninvasive cells were removed with a cotton swab. Images of the glioma cells that had migrated to the other side of the membrane were captured at 100× magnification with a Nikon Eclipse Ti-S microscope (Nikon) with the NIS-Element S.F. software. The numbers of cells were counted in three nonoverlapping, randomly selected fields. In the scratch wound assay, the glioma cells transfected with the appropriate oligonucleotides were incubated for 48 h. A linear wound was made across the cell layer with a 200 μl pipette tip, and three different reference points were marked. The cells were incubated in medium supplemented with 1% FBS for 24 h. After 0 h and 24 h, the wound widths at the same reference points were photographed and measured under a Nikon Eclipse Ti-S microscope (40× magnification; Nikon) with the NIS-Element S.F. software.

### Cell apoptosis assay

Glioma cells were transfected with the appropriate oligonucleotides and incubated for 72 h in an incubator under 5% CO_2_ at 37°C in six-well plates. The cells were harvested with pancreatic enzymes without EDTA and washed twice with precooled PBS. The cells were resuspended at a concentration of 1 × 10^5^ cells/ml in 500 μl of binding buffer containing 5 μl of annexin V-fluorescein isothiocyanate (FITC) and 5 μl of propidium iodide (PI), according to the instructions of the manufacturer of the Annexin V-FITC Apoptosis Detection Kit (KeyGEN, Shanghai, China). After incubation for 15 min at room temperature in the dark, all the samples were analyzed within 1 h with a guava easyCyte 8 flow cytometer (EMD Millipore, USA). The data were analyzed with the De Novo software.

### Statistical analysis

All experiments were performed in triplicate. SPSS version 18.0 (SPSS Software, Chicago, IL, USA) and GraphPad Prism version 5.0 (GraphPad Software, La Jolla, CA, USA) were used for the statistical analyses. Survival curves were constructed with the Kaplan-Meier life table method. The correlation between *HOXB1* and miR-3175 expression was analyzed with Spearman’s rank correlation. Prognostic factors were examined with univariate and multivariate Cox regression models. The data are expressed as means ± SD. Pairwise comparisons were made with *t* tests. *P* < 0.05 was considered statistically significant.

## Results

### *HOXB1* expression is downregulated in glioma

**Fig 1:**
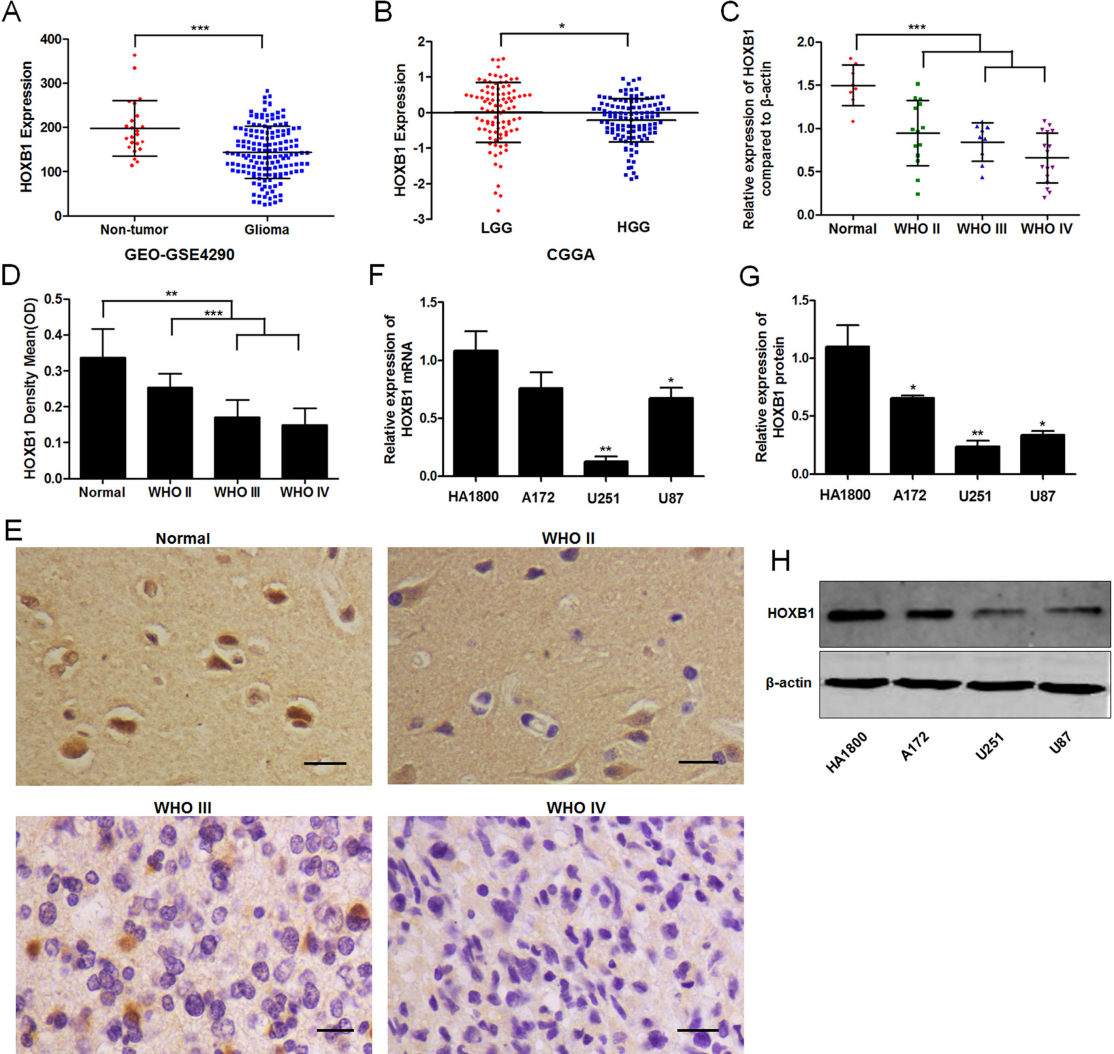
HOXB1 expression in glioma tissues and cell lines. **(A)** HOXB1 expression levels in glioma specimens were analyzed from GEO-GSE4290 data (180 glioma patients). **(B)** HOXB1 expression levels in glioma specimens were analyzed from CGGA data (212 glioma patients). **(C)** HOXB1 mRNA expression in WHO II-IV glioma tissues and non-neoplastic brain tissues were assessed by qRT-PCR. **(D)** HOXB1 protein levels in WHO II-IV glioma tissues and non-neoplastic brain tissues were determined by immunohistochemistry. A statistical analysis is performed as mean ± SD. **(E)** Representative Immunohistochemical staining showing HOXB1 protein levels in non-neoplastic brain tissues and WHO II-IV glioma tissues (400 ×, Scale bars = 20 μm). **(F)** qRT-PCR analysis showed HOXB1 mRNA expression in glioma cell lines A172, U251, and U87. The experiment was repeated three times. **(G-H)** Western blot analysis showed HOXB1 protein expression in glioma cell lines A172, U251, and U87. The experiment was repeated three times, and representative data are shown. (*P < 0.05, **P < 0.01, and ***P < 0.001).

We analyzed and summarized the *HOXB1* expression in whole-gene profiles of human glioma tissues from two independent gene expression data sets, CGGA and GEO. We found that *HOXB1* expression differed significantly between the glioma tissues and nontumor brain tissues, in that *HOXB1* expression was obviously lower in the gliomas (GSE4290; Fig 1A). We also observed that *HOXB1* expression was significantly lower in high-grade glioma tissues (HGG; WHO III and WHO IV) than in low-grade glioma tissues (LGG; WHO I and WHO II) (CGGA; Fig 1B). To further confirm the results of the bioinformatics analysis, qRT-PCR and immunohistochemical analyses were performed on eight nonneoplastic brain tissues and 40 glioma tissues (Fig 1C–1E). The results demonstrated that *HOXB1* expression was markedly lower in glioma tissues than in control tissues. The results also demonstrated that *HOXB1* mRNA expression levels were not visibly different between LGG and HGG in 40 glioma tissues (*P* = 0.069), but the immunohistochemical analyses showed that HOXB1 protein expression levels were clearly downregulated in HGG. HOXB1 expression was also examined in glioma cell lines (A172, U251 and U87). As shown in Fig 1F–1H, the expression of HOXB1 was lower in the glioma cells than in the control HA1800 cells. These results suggest that *HOXB1* functions as a tumor suppressor gene in glioma and that the downregulated expression of *HOXB1* may be related with the degree of malignancy.

### Association of HOXB1 expression with prognosis in glioma patients

**Fig 2:**
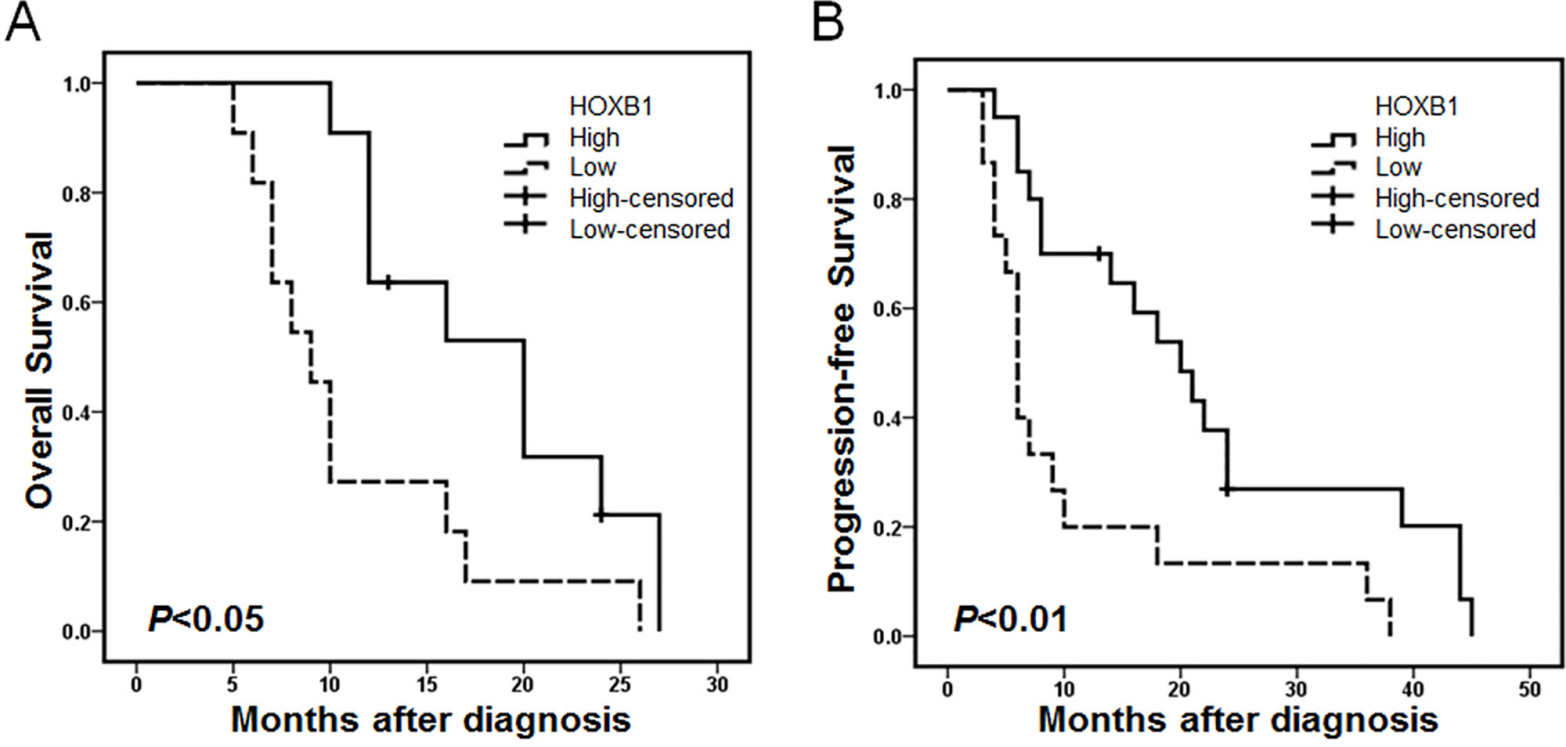
HOXB1 downregulation is associated with poor survival. Kaplan-Meier life table method analysis indicated **(A-B)** that patients with low HOXB1 expression have significantly shorter OS and PFS.

We evaluated the prognostic value of HOXB1 expression for patient OS and PFS based on the follow-up data of glioma patients. OS and PFS were stratified by HOXB1 expression using the Kaplan-Meier life table method and Cox regression models. In this study, we found that high HOXB1 expression predicted longer OS and PFS (Fig 2A and 2B). Univariate Cox regression models showed that only HOXB1 expression (hazards ratio [HR] 0.055, *P* = 0.004, 95% confidence interval [95% CI] 0.008–0.394) was associated with worse survival in this prospective cohort of glioma patients (Table 1). However, glioma grade (HR 2.975, *P* = 0.001, 95% CI 1.702–5.201), and HOXB1 expression (HR 0.111, *P* = 0.001, 95% CI 0.032–0.389) were associated with the progression of glioma. A multivariate analysis showed that HOXB1 expression (HR 0.030, *P* = 0.001, 95% CI 0.004–0.261) and glioma grade (HR 4.682, *P* = 0.021, 95% CI 1.257–17.439) were associated with shorter survival (Table 2). The variables glioma grade (HR 2.732, *P* = 0.001, 95% CI 1.544–5.833) and HOXB1 expression (HR 0.126, *P* = 0.004, 95% CI 0.031–0.511) were associated with PFS. In the Cox regression analysis, the low expression level of HOXB1 was an independent predictor of poor prognosis in glioma patients. These data support the notion that low HOXB1 expression is associated with worse survival in glioma patients and may act as a tumor biomarker in malignant glioma.

**Table 1:** Univariate Cox regression analysis of prognostic factors in glioma patients.

Variable	Univariate analysis					
	OS			PFS		
	HR	95%CI	P	HR	95%CI	P
Gender	0.656	0.248–1.734	0.395	0.645	0.317–1.312	0.226
Age	1.682	0.521–5.425	0.384	2.240	0.798–6.287	0.125
WHO grade	2.989	0.966–9.251	0.057	2.975	1.702–5.201	0.001
KPS	0.564	0.192–1.658	0.298	0.589	0.249–1.392	0.228
Mean tumor diameter ^a^	1.214	0.475–3.105	0.686	0.869	0.426–1.770	0.698
Tumor location ^b^	1.106	0.641–1.909	0.717	1.264	0.729–2.190	0.404
HOXB1 expression	0.055	0.008–0.394	0.004	0.111	0.032–0.389	0.001

^**a**^ Data from imaging measurement.

^**b**^ Patients who suffer from enormous tumor both in frontal lobe, parietal lobe and temporal lobe are counted repeatedly.

**Table 2:** Multivariate Cox regression analysis of prognostic factors in glioma patients.

Variable	Multivariate analysis					
	OS			PFS		
	HR	95%CI	P	HR	95%CI	P
Gender						
Age						
WHO grade	4.682	1.257–17.439	0.021	2.732	1.544–4.833	0.001
KPS						
Mean tumor diameter ^a^						
Tumor location ^b^						
HOXB1 expression	0.030	0.004–0.261	0.001	0.126	0.031–0.511	0.004

^**a**^ Data from imaging measurement.

^**b**^ Patients who suffer from enormous tumor both in frontal lobe, parietal lobe and temporal lobe are counted repeatedly.

### Knockdown of *HOXB1* promotes cell proliferation and invasion, and inhibits apoptosis in glioma cells

**Fig 3:**
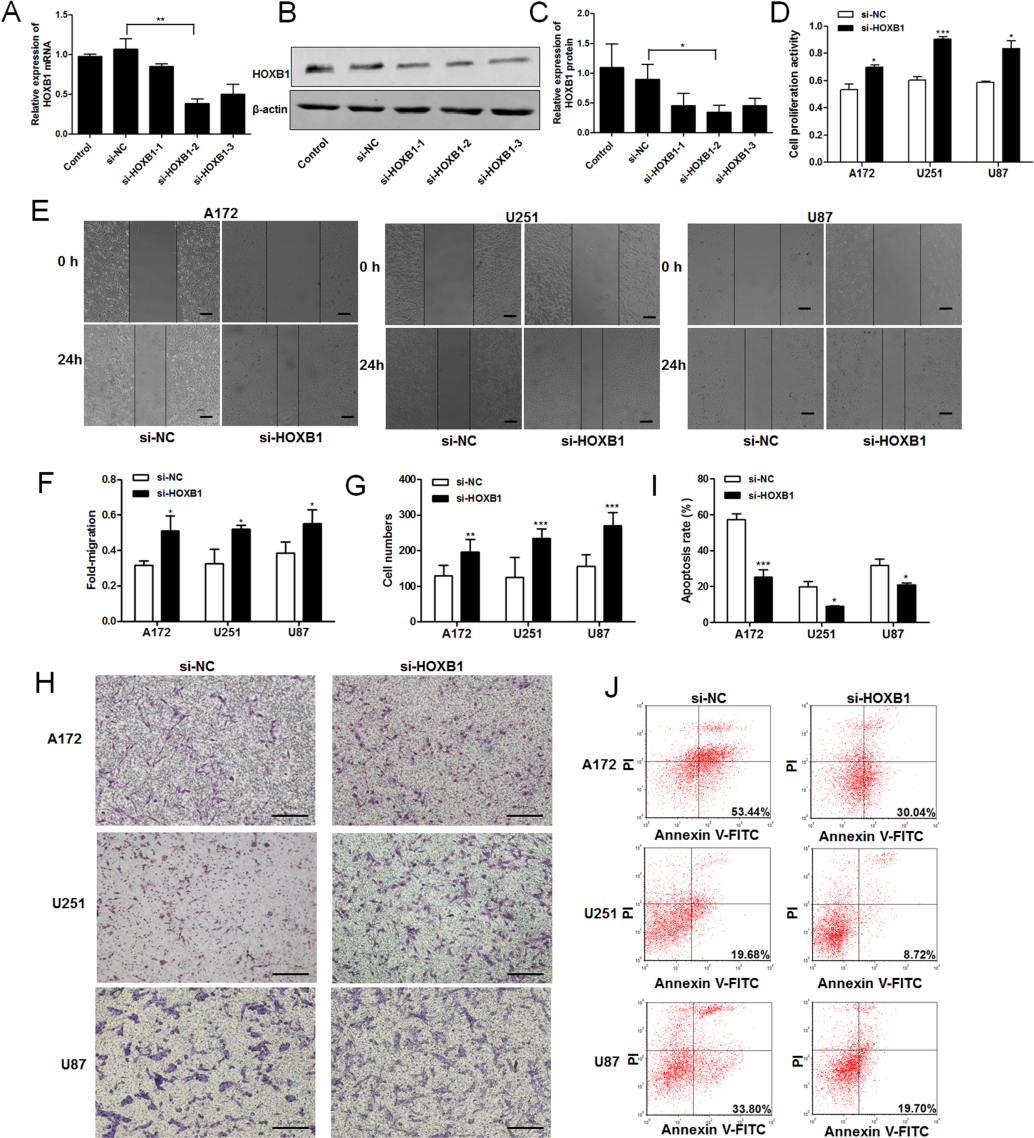
The role of HOXB1 in glioma cell proliferation, invasion, and apoptosis. **(A-C)** Three siRNA targeting different sites of HOXB1 and a siRNA negative control (si-NC) were transfected into U87 cells using Lipofectamine 2000. Total mRNA or total protein was isolated at 24 h or 48 h after transfection. HOXB1 mRNA expression was assessed by qRT-PCR. HOXB1 protein expression was determined with western blot analysis. si-HOXB1-2 as the most efficient siRNA was used in further studies. **(D)** An MTT cell proliferation assay was performed at 48 h after transfection with equal concentrations of si-HOXB1 and si-NC in glioma cell lines. **(E-F)** Scratch wound assay of glioma cell lines transfected with equal concentrations of si-HOXB1 and si-NC. A linear wound was made with a 200 μl pipette tip and the wound width was recorded at three different reference points after 0 and 24 h (40 ×, Scale bars = 200 μm). **(G-H)** Transwell assay of glioma cell lines transfected with equal concentrations of si-HOXB1 and si-NC. The migrated cells were fixed with 4% paraformaldehyde, stained with Giemsa and calculated with microscope in three non-overlapping, randomly selected fields (100 ×, Scale bars = 200 μm). **(I-J)** Flow cytometric analysis of apoptosis in glioma cell lines after transfection with equal concentrations of si-HOXB1 and si-NC. Apoptosis was assessed with Annexin V-FITC/PI. All results are representative of three independent experiments, and a statistical analysis is performed (mean ± SD, *P < 0.05, **P < 0.01, and ***P < 0.001).

To investigate the effects of HOXB1 on glioma cell proliferation, invasion, and apoptosis, we used the most efficient siRNA (si-HOXB1-2) for the targeted knockdown of *HOXB1* expression in glioma cell lines (Fig 3A–3C). A cell proliferation assay showed that the downregulation of *HOXB1* expression significantly promoted cell growth (Fig 3D). The effect of HOXB1 on the invasiveness of glioma cells was investigated with scratch wound and Transwell assays. The extent of wound was healing was greater in glioma cells transfected with si-HOXB1 than in those transfected with si-NC in the scratch wound assay (Fig 3E and 3F). In the Transwell assay, the number of invasive glioma cells increased significantly after they were transfected with si-HOXB1 compared with those transfected with si-NC (Fig 3G and 3H). A cell apoptosis assay showed that the knockdown of *HOXB1* inhibited apoptosis (Fig 3I and 3J). These results suggest that HOXB1 is a tumor suppressor that regulates the proliferation, invasion, and apoptosis of glioma cells in vitro.

### Aberrant expression of miR-3175 in glioma cells affects their proliferation, invasion, and apoptosis

**Fig 4:**
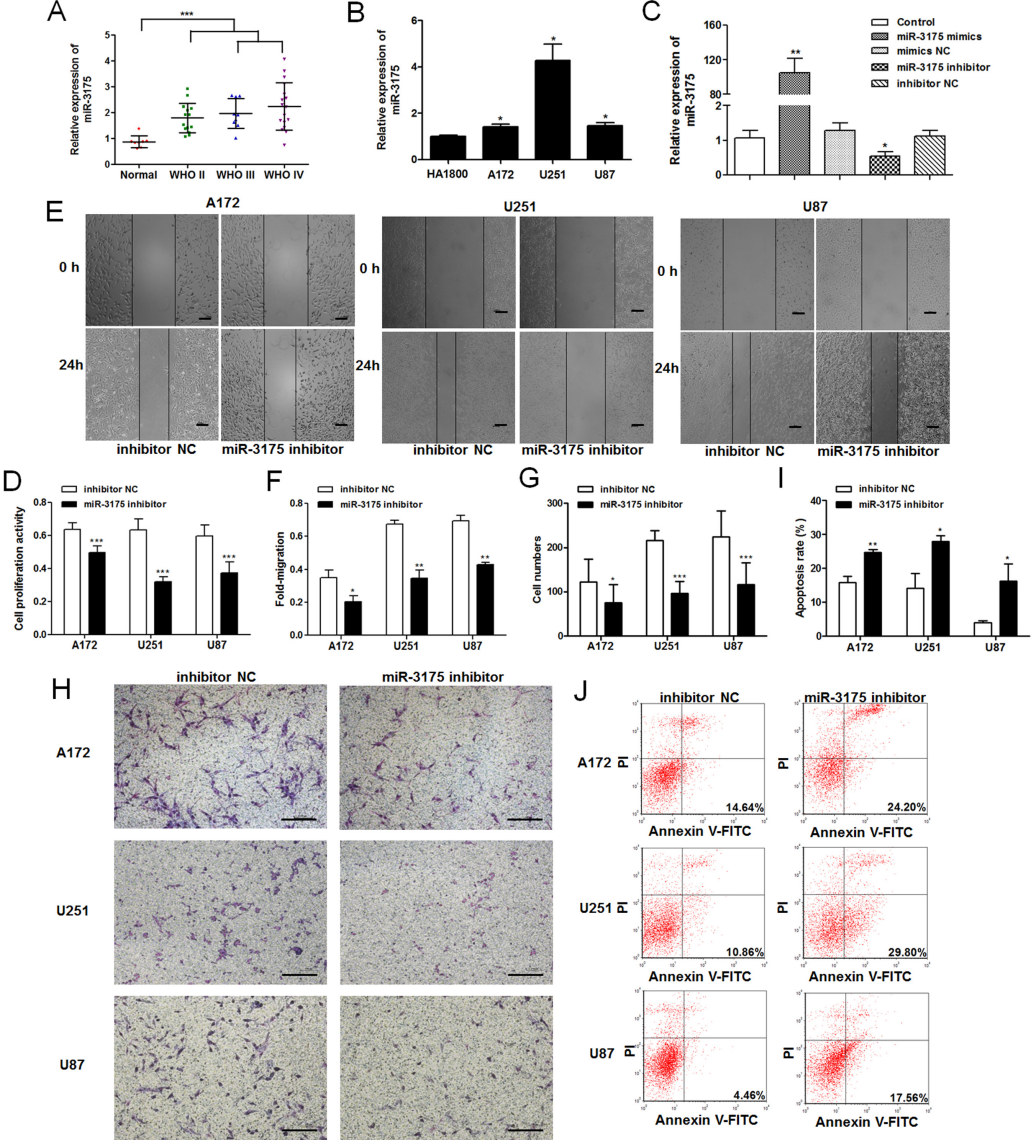
Aberrant expression of miR-3175 in glioma cell lines affects their proliferation, invasion, and apoptosis. **(A)** MiR-3175 expression levels in 40 human glioma tissue specimens and 8 non-neoplastic brain tissue specimens were assessed by qRT-PCR. **(B)** qRT-PCR analysis showed miR-3175 expression in glioma cell lines A172, U251, and U87. **(C)** Equal concentrations of miR-3175 mimics, mimics NC, miR-3175 inhibitor and inhibitor NC were transfected into U87 cells using Lipofectamine 2000. The expression levels of miR-3175 were evaluated by qRT-PCR at 24 h after transfection. **(D)** An MTT cell proliferation assay was performed at 48 h after transfection with equal concentrations of miR-3175 inhibitor and inhibitor NC in glioma cell lines. **(E-F)** Scratch wound assay of glioma cell lines transfected with equal concentrations of miR-3175 inhibitor and inhibitor NC. The wound gaps were photographed and measured at 0 and 24 h (40 ×, Scale bars = 200 μm). **(G-H)** Transwell assay of glioma cell lines transfected with equal concentrations of miR-3175 inhibitor and inhibitor NC. The migrated cells were photographed and counted in three non-overlapping, randomly selected fields (100 ×, Scale bars = 200 μm). **(I-J)** Flow cytometric analysis of apoptosis in glioma cell lines after transfection with equal concentrations of miR-3175 inhibitor and inhibitor NC. Apoptosis was assessed with Annexin V-FITC/PI. All results are representative of three independent experiments, and a statistical analysis is performed (mean ± SD, *P < 0.05, **P < 0.01, and ***P < 0.001).

To assess the role of miR-3175 in glioma, we evaluated the expression levels of miR-3175 in glioma tissues and cell lines with qRT-PCR (Fig 4A and 4B). The results showed that the miR-3175 expression levels were higher in the glioma tissues than in the control tissues, and did not differ significantly between LGG and HGG in 40 glioma tissues (*P* = 0.12). The expression of miR-3175 was elevated in the three glioma cell lines compared with that in the control cells. Because miR-3175 expression is upregulated in glioma, we investigated the biological functions of miR-3175 in glioma. After U87 cells were transfected with the miR-3175 mimic, miR-3175 inhibitor, miR-3175 mimic NC, or miR-3175 inhibitor NC, qRT-PCR was used to determine the expression of miR-3175 (Fig 4C). MiR-3175 expression was significantly increased in the cells transfected with the miR-3175 mimic, but reduced in the cells transfected with the miR-3175 inhibitor, compared with its expression in the corresponding NCs. To study the effects of miR-3175 on cell proliferation, invasion, and apoptosis, we reduced the expression of miR-3175 by transfecting cells with the miR-3175 inhibitor. A cell proliferation assay showed that the growth of the A172, U251, and U87 cells was clearly inhibited when they were transfected with the miR-3175 inhibitor compared the growth of those transfected with the inhibitor NC (Fig 4D). The invasive behavior of glioma cells after their transfection with the miR-3175 inhibitor or inhibitor NC indicated that the reduced expression of miR-3175 markedly suppresses the invasiveness of glioma cells (Fig 4E–4H). In the cell apoptosis assay, cells transfected with the miR-3175 inhibitor showed a clearly higher rate of apoptosis than cells transfected with the inhibitor NC (Fig 4I and 4J).

### *HOXB1* is a direct target of miR-3175

**Fig 5:**
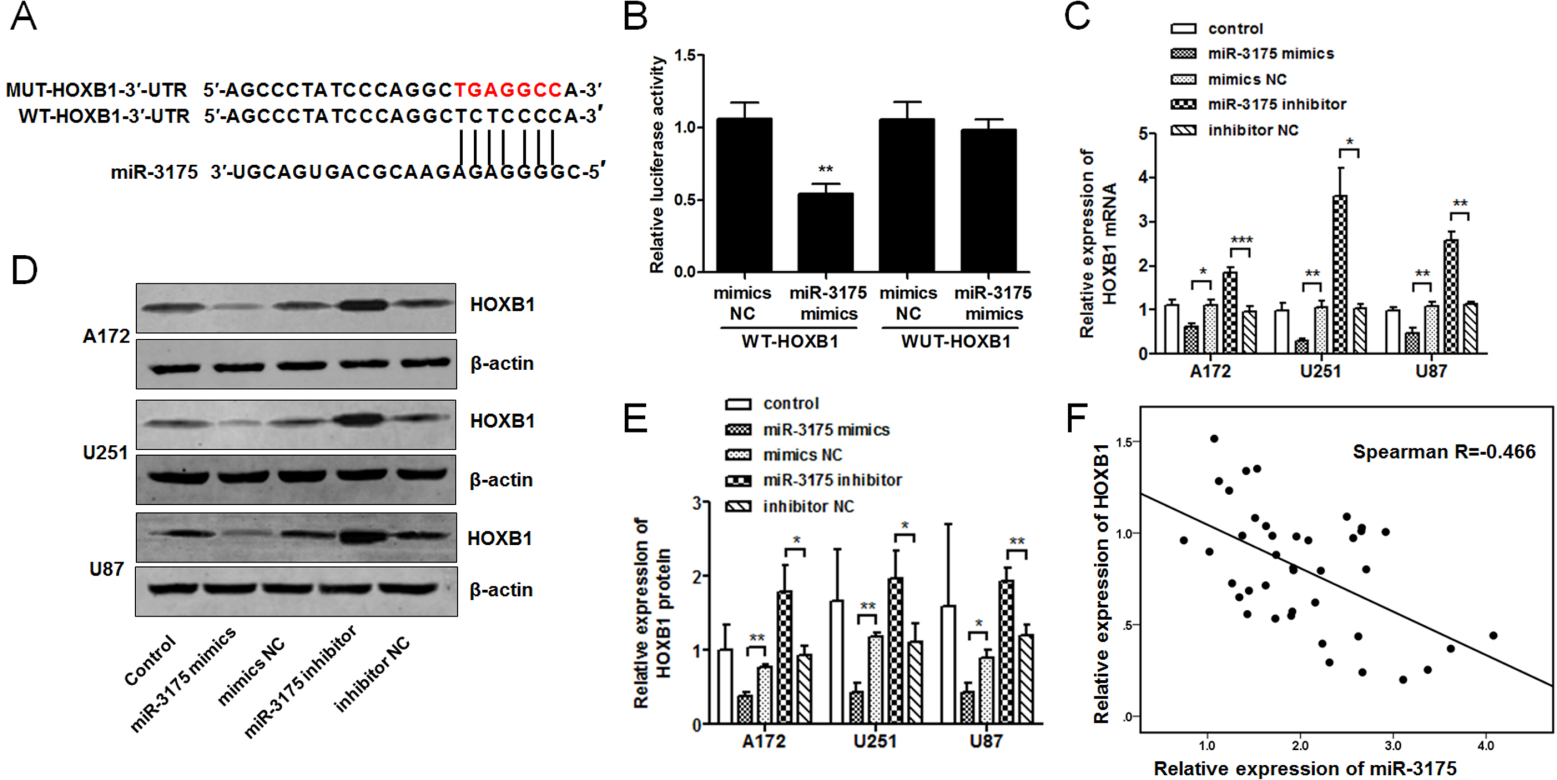
HOXB1 is a direct target of miR-3175. **(A)** Diagram of the seed sequence of the miR-3175-binding site within the wild-type HOXB1 3′-UTR and the design of the mutated HOXB1 3′-UTR sequence. The mutated HOXB1 3′-UTR sequence is labeled in red. **(B)** Luciferase assay of U87 cells after cotransfection with the wild-type or mutant HOXB1 3′-UTR and miR-3175 mimics or miR-3175 mimics NC. The pRL-TK Renilla luciferase reporter vector was used as the internal control. Luciferase activity = firefly luciferase/Renilla luciferase. **(C)** HOXB1 mRNA expression is modulated by miR-3175. Glioma cell lines were transfected with miR-3175 mimics, miR-3175 inhibitor and the corresponding NCs, and incubated for 72 h. Cells were harvested and assayed using qRT-PCR. **(D-E)** Western blot analysis of HOXB1 protein levels in glioma cell lines after transfection with miR-3175 mimics, miR-3175 inhibitor and the corresponding NCs at 72 h. **(F)** The correlation between HOXB1 and miR-3175 expression in human glioma tissue specimens was analyzed with Spearman’s rank correlation. All results are representative of three independent experiments, and a statistical analysis is performed (mean ± SD, *P < 0.05, **P < 0.01, and ***P < 0.001).

To ascertain the mechanisms by which miR-3175 regulates *HOXB1* expression through binding sites in the *HOXB1* 3′-UTR, as predicted in the bioinformatics analysis (Fig 5A), we synthesized the pmirGLO-WT-HOXB1 and pmirGLO-MUT-HOXB1 plasmids, and performed a *HOXB1* 3′-UTR luciferase reporter assay in U87 cells. The luciferase activity was significantly reduced in the glioma cells transfected with the miR-3175 mimic and pmirGLO-WT-HOXB1, but no reduction was observed in the cells transfected with pmirGLO-MUT-HOXB1 (Fig 5B). These results suggest that *HOXB1* is a direct target of miR-3175. To confirm the relationship between *HOXB1* and miR-3175, qRT-PCR and western blot analysis were used to investigate the expression levels of HOXB1 in glioma cell lines 72 h after transfection with miR-3175 mimic, miR-3175 inhibitor, or the corresponding NCs. As shown in Fig 5C–5E, the HOXB1 expression levels in the glioma cell lines were significantly reduced after transfection with the miR-3175 mimic, and increased after transfection with the miR-3175 inhibitor. Spearman’s rank correlation analysis showed that the expression levels of *HOXB1* and miR-3175 were inversely correlated in 40 human tissues (Spearman’s R = -0.466; Fig 5F).

### Oncogenicity induced by low *HOXB1* expression is prevented by miR-3175 inhibitor in glioma cells

**Fig 6:**
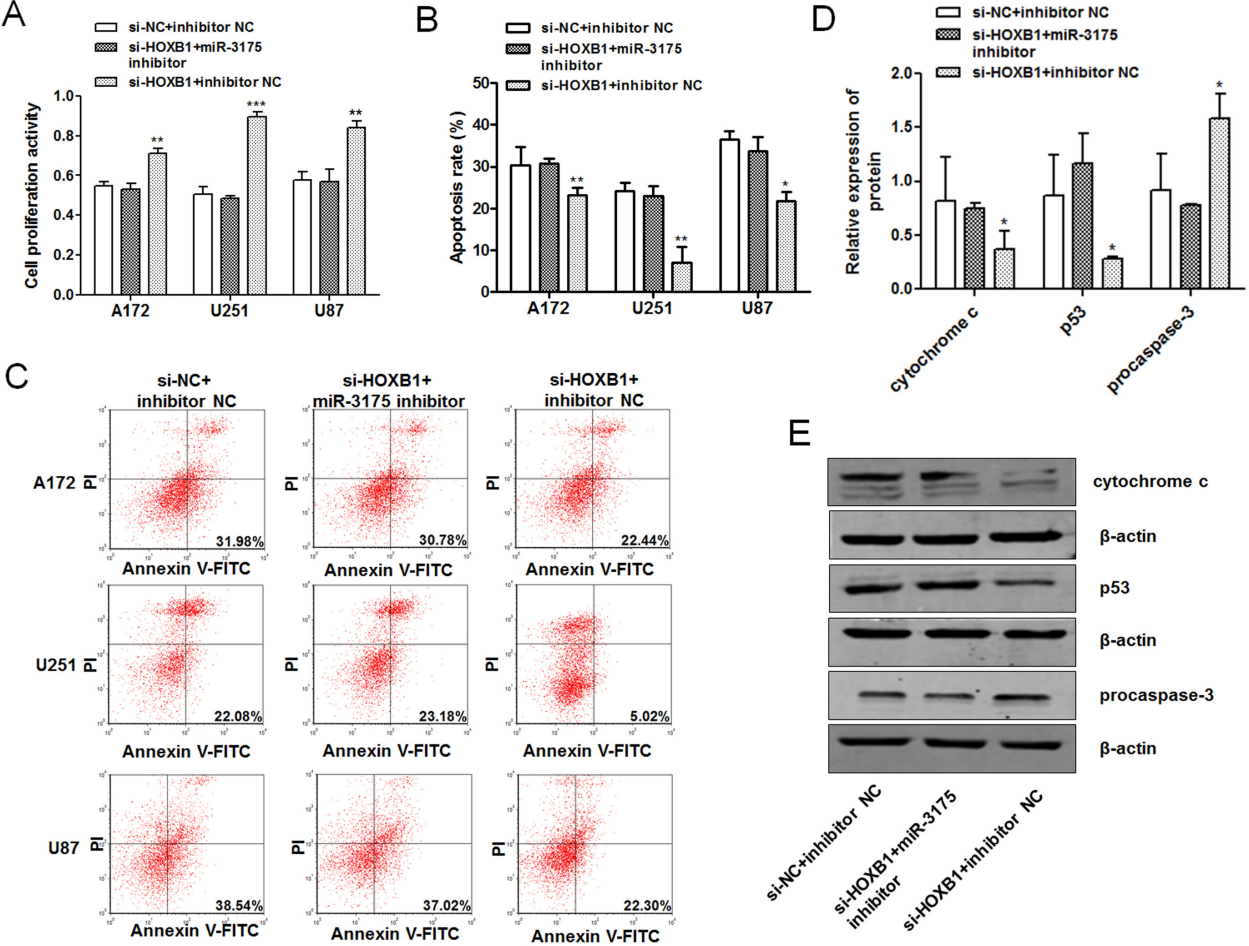
Oncogenicity induced by low HOXB1 expression is prevented by miR-3175 inhibitor in glioma cells. **(A)** An MTT cell proliferation assay was performed at 48 h after transfection with si-NC + inhibitor NC, si-HOXB1 + miR-3175 inhibitor and si-HOXB1 + inhibitor NC in glioma cell lines. **(B-C)** Flow cytometric analysis of apoptosis in glioma cell lines after transfection with si-NC + inhibitor NC, si-HOXB1 + miR-3175 inhibitor and si-HOXB1 + inhibitor NC. Apoptosis was assessed with Annexin V-FITC/PI. **(D-E)** Western blot analysis of several apoptosis-related proteins (procaspase-3, p53 and cytochrome c) expression in U87 cells after transfection with si-NC + inhibitor NC, si-HOXB1 + miR-3175 inhibitor and si-HOXB1 + inhibitor NC. All results are representative of three independent experiments, and a statistical analysis is performed (mean ± SD, *P < 0.05, **P < 0.01, and ***P < 0.001).

As shown above, we found that *HOXB1* is a direct target of miR-3175 and *HOXB1* expression was significantly reduced after transfection with the miR-3175 mimic. We also demonstrated that the reduced expression of HOXB1 promoted cell proliferation and invasion and inhibited apoptosis, similar to the phenomena induced by miR-3175. The miR-3175 inhibitor was shown to inhibit the proliferation and invasion of glioma cells and to promote cell apoptosis in glioma. Cell proliferation and apoptosis assays were performed to clarify whether the oncogenicity induced by low HOXB1 expression was affected by miR-3175 in glioma, after cells were cotransfected with si-HOXB1, the miR-3175 inhibitor, or the corresponding NCs. The results showed that the miR-3175 inhibitor significantly prevented the increased cell proliferation and suppressed the apoptosis induced by low HOXB1 expression (Fig 6A–6C), indicating strongly that miR-3175 is a critical participant in the tumor-suppressor activity of HOXB1 in glioma. A western blot analysis of several downstream apoptosis-related proteins (procaspase-3, p53, and cytochrome c) was performed to preliminarily investigate the signaling pathway affecting HOXB1-induced apoptosis, which is dysregulated in the pathogenesis of glioma. This analysis showed that procaspase-3, p53, and cytochrome c are involved in HOXB1-induced apoptosis (Fig 6D and 6E).

## Discussion

It is well known that the expression products of the HOX family genes have DNA-binding activity and act as transcription factors. Functioning as both positive and negative regulatory factors, HOX proteins not only control the proliferation and differentiation of embryonic cells, but also have important functions in the differentiation of adult cells [17–18]. The dysregulation of HOX gene expression leads to abnormal morphological structures during the growth of individuals, and can even cause tumorigenesis, including glioma, by altering the expression of apoptosis-related proteins and/or signaling pathways [19–20]. The upregulation of HOXA9 [21], HOXC6, HOXC11 [22], HOXD1, and HOXD8 [23] expression is associated with the proliferation and growth of glioma. Although recent studies have reported that HOXB1 plays a key role in the tumorigenesis of many kinds of cancer [9–12], and that its expression correlates with oriented cell division in the neural tube [24] and embryonal carcinoma cell differentiation stimulated with retinoic acid [25–26], the function of HOXB1 in glioma is still unclear. We investigated the activity and molecular mechanism of HOXB1 in modulating the pathogenesis of glioma. In this study, we determined HOXB1 expression in 40 glioma tissues and three glioma cell lines, and showed that HOXB1 expression is significantly downregulated in glioma compared with its expression in the corresponding controls. Although we found that the expression of *HOXB1* mRNA in HGG did not differ significantly from that in LGG, which may be attributable to the small sample sizes, the results of immunohistochemical and bioinformatics analyses suggest that the downregulation of HOXB1 may be associated with a greater degree of malignancy. The oncogenicity induced by the downregulated expression of HOXB1 in glioma involves the promotion of cell proliferation, enhanced cell invasion, and the inhibition of apoptosis in vitro. Based on the clinical features of our patients, this study provides significant evidence that the downregulation of HOXB1 is associated with worse survival and that the level of HOXB1 expression is an independent predictor of prognosis in glioma patients. Therefore, this study of the molecular mechanism of HOXB1 function in glioma should significantly improve the diagnosis and treatment of this malignancy.

MiRNAs are a class of small endogenous noncoding regulatory RNAs that negatively regulate gene expression at the posttranscriptional and/or translational level by binding to the incomplete complementary strand of the mRNA 3'-UTR [27]. The miRNAs that regulate the HOX gene clusters are conserved between *Drosophila melanogaster* and humans [28]. MiRNAs are traditionally believed to regulate HOX gene expression by mRNA cleavage or by interrupting the association between the mRNA and the translation machinery [29]. Accumulated evidence has strongly implicated abnormally expressed HOX genes, caused by the miRNA regulation of their 3′-UTRs, in a wide variety of human cancers. For instance, HOXD10 and miR-146a are associated with head and neck squamous cell carcinomas [30], and HOXA1, under the regulation of miR-100, in small-cell lung cancer [31].

Our knowledge of the mechanism that regulates HOXB1 in cancer has predominantly involved the three-amino-acid-loop-extension (TALE) homeodomain factors that act synergistically with HOX [32], and it was unclear whether upstream miRNAs regulate HOXB1 expression in glioma. Using a computer-assisted bioinformatics analysis, we predicted that miR-3175 directly targets and suppresses *HOXB1* expression. A recent study found that miR-3175 acts as a tumor suppressor in gastric cancer [33], and microarray profiling demonstrated its association with chronic congestive heart failure [34]. However, little is known about the effects of miR-3175 on other kinds of human cancer, including glioma. In this study, the expression of miR-3175 was analyzed in glioma tissues and cell lines with qRT-PCR. The results clearly indicate that miR-3175 expression is elevated in glioma, and the downregulation of miR-3175 significantly suppressed glioma cell proliferation and invasion, and promoted their apoptosis.

This finding supports a role for miR-3175 as a tumor promoter in glioma and raises the possibility that miR-3175 regulates *HOXB1* expression. In this study, *HOXB1* was shown experimentally to be a novel target of miR-3175. A luciferase reporter assay confirmed that miR-3175 directly recognizes the 3′-UTR of *HOXB1* transcripts. An miR-3175 mimic downregulated *HOXB1* expression and an miR-3175 inhibitor upregulated it, demonstrating that the expression of both *HOXB1* mRNA and HOXB1 protein are significantly regulated by miR-3175 in glioma cell lines. The inverse correlation between HOXB1 expression and miR-3175 expression was confirmed in clinical specimens with Spearman’s rank correlation. A cotransfection analysis was performed to verify that the tumorigenesis induced by the reduced expression of HOXB1 in glioma is regulated by miR-3175. The oncogenicity induced in glioma cells by low HOXB1 expression was not only reversed by an miR-3175 inhibitor, evident as reduced cell proliferation and increased cell apoptosis, but the expression of several apoptosis-related proteins was affected by low HOXB1, including that of procaspase-3, p53, and cytochrome c. Procaspase-3 expression was enhanced and cytochrome c expression was reduced in cells in which HOXB1 expression was downregulated. Therefore, we suggest that HOXB1 triggers both the mitochondrial and caspase-dependent apoptotic pathways [35].

The expression of HSP70 interacting protein (ST13), c-Jun N-terminal kinase 2 (JNK2), and programmed cell death 10 (PDCD10) is HOXB1-dependently upregulated, and these proteins are involved in mitochondrial permeabilization and the induction of apoptosis [10]. The HOXB1 protein forms complexes with TALE cofactors that control the expression of various genes, including cAMP response element binding protein (*CBP*) [36], and CBP-dependent protein histone acetyltransferase (HAT) is associated with the p53 signaling pathway [37]. The HOXB1-dependently downregulated gene, mouse double minute 2 homolog (*MDM2*), is also involved in p53-induced apoptosis [10]. PBX1 is a cofactor of HOXB1 that reportedly regulates p53, in cooperation with PREP1 [38–39], a TALE protein, and activates and modulates the expression of p63, another member of the p53 family [40]. More importantly, HOXB1 enhances the capacity of all-trans retinoic acid (ATRA) to induce apoptosis [10], and ATRA is reported to upregulate the expression of p53 and to inhibit the proliferation of glioma cells [41]. In this study, p53 expression was significantly reduced in cells in which HOXB1 expression was downregulated. Consequently, the p53 signaling pathway might play an important role in HOXB1-induced apoptosis.

In conclusion, our data suggest that *HOXB1* is a tumor suppressor gene in glioma. The downregulation of HOXB1 expression promotes glioma cell proliferation and invasion and inhibits cell apoptosis. More importantly, *HOXB1* was shown experimentally in this study to be a direct target of miR-3175. Although much remains to be investigated regarding the role of HOXB1 in the pathogenesis of glioma, as a novel biomarker, HOXB1 represents a potential target for the diagnosis and treatment of glioma.

## Supporting Information

S1 Dataset The whole western blot images in vitro. **(A)** The whole western blot images show HOXB1 protein expression in glioma cell lines A172, U251, and U87. **(B)** The whole western blot images show HOXB1 protein expression in U87 cells after transfection with three siRNA targeting different sites of HOXB1 and a siRNA negative control at 48 h. **(C)** The whole western blot images show HOXB1 protein expression levels in glioma cell lines after transfection with miR-3175 mimics, miR-3175 inhibitor and the corresponding NCs at 72 h. **(D)** The whole western blot images show several apoptosis-related proteins (procaspase-3, p53 and cytochrome C) expression in U87 cells after transfection with si-NC + inhibitor NC, si-HOXB1 + miR-3175 inhibitor and si-HOXB1 + inhibitor NC.
(RAR)
